# Mutation analysis of the Fanconi anaemia A gene in breast tumours with loss of heterozygosity at 16q24.3

**DOI:** 10.1038/sj.bjc.6690168

**Published:** 1999-03

**Authors:** A-M Cleton-Jansen, E W Moerland, J C Pronk, C G M van Berkel, S Apostolou, J Crawford, A Savoia, A D Auerbach, C G Mathew, D F Callen, C J Cornelisse

**Affiliations:** Department of Pathology, Leiden University Medical Center, The Netherlands; Department of Human Genetics, Free University Amsterdam, The Netherlands; Department of Cytogenetics and Molecular Genetics, Adelaide's Women and Children's Hospital, North Adelaide, Australia; Ospedale Casa Sollievo della Sofferenza, Foggia, Italy; Laboratory of Human Genetics and Hematology, The Rockefeller University, New York, NY, USA; Division of Medical and Molecular Genetics UMDS, Guy's Hospital, London, UK

**Keywords:** Fanconi anaemia, breast cancer, allelic imbalance, tumour suppressor gene, chromosome 16

## Abstract

The recently identified Fanconi anaemia A (*FAA*) gene is located on chromosomal band 16q24.3 within a region that has been frequently reported to show loss of heterozygosity (LOH) in breast cancer. *FAA* mutation analysis of 19 breast tumours with specific LOH at 16q24.3 was performed. Single-stranded conformational polymorphism (SSCP) analysis on cDNA and genomic DNA, and Southern blotting failed to identify any tumour-specific mutations. Five polymorphisms were identified, but frequencies of occurrence did not deviate from those in a normal control population. Therefore, the *FAA* gene is not the gene targeted by LOH at 16q24.3 in breast cancer. Another tumour suppressor gene in this chromosomal region remains to be identified. © 1999 Cancer Research Campaign


*British Journal of Cancer* (1999) **79**(7/8), 1049–1052
© 1999 Cancer Research Campaign
Article no. bjoc.1998.0168

### Mutation analysis of the Fanconi anaemia A gene in breast tumours with loss of heterozygosity at 16q24.3

**A-M Cleton-Jansen[1], EW Moerland[1], JC Pronk[2], CGM van Berkel[2], S Apostolou[3], J Crawford[3], A Savoia[4], AD Auerbach[5], CG Mathew[6], DF Callen[3] and CJ Cornelisse[1]**

[1]Department of Pathology, Leiden University Medical Center, The Netherlands; [2]Department of Human Genetics, Free University Amsterdam, The Netherlands; [3]Department of Cytogenetics and Molecular Genetics, Adelaide's Women and Children's Hospital, North Adelaide, Australia; Ospedale Casa Sollievo della Sofferenza, Foggia, Italy; [5]Laboratory of Human Genetics and Hematology, The Rockefeller University, New York, NY, USA; [6]Division of Medical and Molecular Genetics UMDS, Guy's Hospital, London, UK

**Summary** The recently identified Fanconi anaemia A (*FAA*) gene is located on chromosomal band 16q24.3 within a region that has been frequently reported to show loss of heterozygosity (LOH) in breast cancer. *FAA* mutation analysis of 19 breast tumours with specific LOH at 16q24.3 was performed. Single-stranded conformational polymorphism (SSCP) analysis on cDNA and genomic DNA, and Southern blotting failed to identify any tumour-specific mutations. Five polymorphisms were identified, but frequencies of occurrence did not deviate from those in a normal control population. Therefore, the *FAA* gene is not the gene targeted by LOH at 16q24.3 in breast cancer. Another tumour suppressor gene in this chromosomal region remains to be identified.

**Keywords:** Fanconi anaemia; breast cancer; allelic imbalance; tumour suppressor gene; chromosome 16

Tumour suppressor genes are characterized by mutational inactivation of both alleles in tumour DNA. In general, one copy is inactivated by a small mutation, i.e. a point mutation or frameshift, whereas the other copy is lost by large genomic deletions or mitotic recombination, reflected in loss of heterozygosity (LOH) of adjacent polymorphic markers. LOH on the long arm of chromosome 16 has been detected frequently in breast tumours (Cleton-Jansen et al, 1994; Tsuda et al, 1994; Dorion-Bonnet et al, 1995; Skirnisdottir et al, 1995; Iida et al, 1997), and also other tumours, especially prostate cancer (Cher et al, 1995; Suzuki et al, 1996; Latil et al, 1997). Detailed deletion mapping has shown that several regions on chromosome arm 16q are targets for LOH, one of which is located near the telomere at 16q24.3. This region is bordered by markers *APRT* and D16S303.

A strong candidate gene located within this region is the Fanconi Anaemia group A (*FAA*) gene, which was recently identified (Pronk et al, 1995; Fanconi Anemia/Breast Cancer Consortium, 1996; Lo Ten Foe et al, 1996). *FAA* encodes a protein of 1455 amino acids and has no significant homology to any other protein. Fanconi anaemia (FA) is an autosomal recessive genetic disorder characterized by progressive pancytopenia and congenital malformations. Since FA patients have a high predisposition to develop malignancies, and their cells show chromosomal instability and increased sensitivity to bifunctional alkylating agents in vitro, we postulated that the *FAA* gene could be the gene targeted by LOH on 16q24.3.

*Received 30 January 1998*
*Revised 4 August 1998*
*Accepted 18 August 1998*

*Correspondence to:* A-M Cleton-Jansen, Department of Pathology, Leiden University Medical Center, PO Box 9600, NL-2300 RC Leiden, The Netherlands

This report describes a mutation analysis of the retained copy of the *FAA* gene in breast tumours with LOH only at 16q24.3. This specific set of tumours ensures that the gene at 16q24.3 is the target for LOH and not another gene on chromosome 16q, e.g. a gene at 16q22.1, a region also implicated in LOH.

### MATERIALS AND METHODS

### Tumour material

Freshly frozen breast tumour tissue was collected at the Department of Pathology of the Leiden University Medical Center. Haematoxylin and eosin-stained sections of tumour tissue blocks were examined by a pathologist to select cases that contained at least 50% tumour cells. Tumours for *FAA* mutation analysis were selected by testing polymorphic markers on chromosome 16 as has been described previously (Cleton-Jansen et al, 1994). Seventeen tumours showing LOH only on chromosomal band 16q24.3, two tumours showing complex LOH on 16q22 and 16q24.3 but retention of markers in between and three controls without 16q LOH were used in this study. The LOH pattern on 16q of the tumours was shown by dense LOH mapping, which was described in detail by Moerland et al (1997). Genomic DNA from 50 healthy controls originating from the Dutch population was used for testing frequencies of polymorphisms.

### Mutation screening

RNA was extracted from 10–20 sections of 20 $\mu$m from tumour tissue blocks using TRIzol reagent (GIBCO BRL) according to the manufacturer's instructions. cDNA was synthesized from 1 to 10 $\mu$g RNA in a 20 $\mu$l reaction volume containing 0.2 units AMV reverse transcriptase (RT; Boehringer), 1 $\mu$g random primed hexamers, 2 mM dNTP in the buffer supplied with the enzyme.

**Table 1** SSCP analysis of FAA on breast tumour cDNA

| cDNA<br>fragment<br>exon | *FAA1*<br>–7–400<br>1–4 | *FAA2*<br>335–719<br>4–7 | *FAA3*<br>656–1368<br>8–14 | *FAA4*<br>1323–1684<br>15–18 | *FAA5*<br>1625–2011<br>18–22 | *FAA6*<br>1924–2558<br>22–27 | *FAA7*<br>2017–2826<br>23–29 | *FAA8*<br>2783–4408<br>29–43 |
|---|---|---|---|---|---|---|---|---|
| *Tumours with LOH only on 16q24* | | | | | | | | |
| BT309 | N[b] | N | 796AG[c] | 1501AG | N | 2426AA | | |
| BT358 G[a] | [d] | | 796AG | 1501AG | | | | |
| BT367 | | N | 796AG | 1501AG | N | 2426AG | | |
| BT378 | N | N | | | N | | | |
| BT408 | N | | 796GG | 1501AG | N | 2426AA | | |
| BT410 | N | N | 796AA | 1501AG | N | 2426GG | | |
| BT413 | N | N | 796AG | 1501AG | | 2426AA | N | N |
| BT465 | | | 796AA | 1501GG | | 2426AG | | |
| BT470 | N | N | 796AA | 1501GG | | 2426AG | | |
| BT541 G | N | N | 796AG | 1501AG | N | 2426GG | N | N |
| BT559 G | N | N | 796AG | 1501AG | N | 2426AG | N | N |
| BT589 G | N | N | 796GG | 1501GG | | 2426AA | | |
| BT666 G | | | 796AG | 1501AG | N | 2426GG | | |
| BT757 G | | N | 796AG | 1501AA | N | 2426AA | | |
| BT819 | N | N | 796AA | 1501GG | N | 2426GG | N | N |
| BT912 | N | N | 796AA | 1501GG | | | | |
| BT919 G | | N | 796GG | 1501AG | | 2426AA | N | N |
| *Tumours with complex LOH only on 16q24 and 16q22* | | | | | | | | |
| BT355 | N | N | 796GG | 1501GG | N | 2426GG | N | N |
| BT555 G | | N | 796GG | 1501AG | N | 2426GG | N | N |
| *Tumours with retention on chromosome 16q* | | | | | | | | |
| BT335 | | | | 1501AG | | 2426AA | | |
| BT655 | | N | 796GG | 1501GG | | 2426GG | | |
| BT805 | N | N | 796GG | 1501AG | N | 2426AA | | |

[a]G indicates that this sample was also analysed in a genomic DNA based exon-by-exon SSCP. [b]N indicates that the sample was tested, but no variant bands were found. [c]Numbers refer to the position of a variant nucleotide in the cDNA sequence (1 = first basepair of the FAA start codon); letters indicate which alleles are identified in the sample.[d]Empty cells: sample not tested for this fragment.

Polymerase chain reaction (PCR) reactions were performed on 2 μl cDNA with primers derived from the *FAA* cDNA sequence. Table 1 shows the PCR strategy. Primer sequences and PCR conditions are available on request. PCR fragments *FAA*1 to *FAA*6 were radiolabeled by the addition of 2 μCi $\alpha$-$^{32}$P-dCTP and digested with restriction enzymes resulting in fragments of appropriate length to be analysed by single-strand conformational polymorphism (SSCP). *FAA*7 and *FAA*8 were amplified in a first step, followed by a nested PCR, which resulted in 3 and 6 radiolabelled fragments, respectively, with a size range between 200 and 400 bp.

RT-PCR products were analysed on SSCP gels containing 10% glycerol, 6% acrylamide and TBE or MDE gels (Boehringer). If a variant band pattern was observed in an RT-PCR fragment, sequencing was performed using a Cycle Sequencing kit (Perkin Elmer). Subsequently, restriction enzyme analysis on PCR products amplified from genomic DNA of tumour and normal tissue of all patients and on DNA from healthy controls was applied to determine the frequency of the variant alleles (Table 2).

Genomic DNA from eight tumours was also tested in an exon-by-exon SSCP analysis described elsewhere (Wijker et al, 1998). The 43 exons of *FAA* (Lanzano et al, 1997) were amplified from genomic DNA with primers located 40–60 bp from the exon boundaries with T7 (sense) and Sp6 (antisense) sequences to facilitate sequencing. Fragments were analysed by SSCP on a 20% acrylamide gel with silver staining to visualize the bands. Southern analysis to identify genomic rearrangements or large deletions was performed as described previously (Devilee et al, 1991).

### RESULTS

Table 1 shows the results of the SSCP analysis on cDNA of the tumours. No tumour-specific variations were found, either in cDNA or genomic DNA. SSCP analysis sometimes resulted in altered banding patterns. Upon sequencing of the PCR fragments showing variations, these always appeared to be polymorphisms of the *FAA* gene that could also be identified in a normal control population. Moreover, analysis of DNA from non-neoplastic cells of the same patient showed that these variants were also present in the germline and were, therefore, not tumour-specific. Both alleles of a polymorphism could be recognized in DNA from tumour tissue, due to a 20–50% contamination with non-neoplastic cells. Table 2 shows these polymorphisms, their location, nature, result on the amino acid sequence, and occurrence in breast tumours and in controls. Although all four polymorphisms resulted in an amino acid change, none of these appeared to be more predominant in patients with breast cancer and cannot, therefore, be considered as pathogenic germline mutations. An exception is the 17T/A (V/D6) variant, which was found in only 1 out of 100 chromosomes of the control population but in 2 out of 16 chromosomes in patients. However, in one patient, case BT555, there was LOH of the 17T allele, whereas in the other, case BT757, the 17A allele showed LOH, indicating that there is no preferential LOH for one allele or the other. This polymorphism was also identified in a Fanconi anaemia A family, but did not segregate with the disease phenotype (Levran et al, 1997). The V/D6 variant was only detected by

*© Cancer Research Campaign 1999*

**Table 2** Polymorphisms detected in FAA in breast tumour samples

| Location | Exon | Variation | AA change | % in breast cancer[a] | % in controls[a] | Restriction enzyme[b] |
|---|---|---|---|---|---|---|
| 17 | 1 | T/A | V/D6 | 12% [16] | 1% [100] | Destroys *DrdI* |
| 796 | 9 | A/G | T/A266 | 47% [34] | 50% [50] | Creates *BstUI* |
| 1501 | 16 | G/A | G/S501 | 65% [38] | 65% [94] | Destroys *MspI* |
| 2426 | 26 | G/A | G/D809 | 50% [38] | 46% [60] | Destroys *DraIII* |

[a]% of variant allele; between brackets: number of chromosomes tested. [b]Restriction enzyme analysis for detection of the polymorphism in PCR fragments on genomic DNA.

SSCP analysis on genomic DNA, not on cDNA, most probably because the PCR primer for the genomic assay was located 50 bp upstream of the start codon, and the cDNA primer was only 7 bp upstream.

Evidence for large genomic deletions in FA patients has been reported (Fanconi Anemia/Breast Cancer Consortium, 1996). Since large deletions and other rearrangements cannot be detected by PCR a subset of seven tumours with LOH at 16q24.3, six breast tumour cell lines and two normal mammary epithelial cell lines, were examined by Southern analysis. DNA from breast tumour cell lines was obtained from CAMA-1, MCF7, MDA468, BT474, ZR75-1 and MPE600 (kindly provided by Dr Joe Gray), and from SV40 transformed mammary epithelial cell lines HBL100 and RC6. Four of these breast tumour cell lines show homozygosity for four highly polymorphic markers near the *FAA* gene, suggesting that LOH had occurred at 16q24. DNA was digested with *MspI*, *TaqI*or *PvvuII*, blotted onto Hybond N+ membranes and hybridized with an *FAA* full-length cDNA probe (provided by Dr Hans Joenje). None of the tumour samples or cell lines showed aberrant bands. The *FAA* cDNA recognized a variant band of 3.4 kb in *PvuII* digests of two out of seven tumours tested, but this was also present in non-neoplastic DNA and is, therefore, considered to be a polymorphism. Southern analysis of DNA from 11 healthy controls showed that this polymorphism could be found in two individuals, a frequency similar to that in patients.

### DISCUSSION

The most distal region of chromosome arm 16q has been frequently implicated as a target for LOH in both breast cancer and prostate cancer and is, therefore, likely to contain a tumour suppressor gene. LOH studies of this chromosome arm in breast tumours have also implicated other candidate regions. The E-cadherin gene located at 16q22.1 was actually shown to be the target of LOH, i.e. one copy was affected by a protein truncating mutation and the other copy removed by LOH (Berx et al, 1995, 1996). The E-cadherin gene, however, is only targeted in lobular breast tumours, which comprise a minority of 5–10% of the total number of cases.

The Fanconi anaemia A susceptibility gene is located at 16q24.3 (Pronk et al, 1995; Gschwend et al, 1996) in the smallest region of overlap that was defined by detailed deletion mapping of 79 breast tumours (Cleton-Jansen et al, 1994). *FAA* encodes a unique protein that does not exhibit any homology to known proteins that might suggest a function. Recently, it was suggested that FA is due to a defective caretaker gene, as are ataxia-telangiectasia, Bloom syndrome, hereditary non-polyposis colorectal cancer, Werner syndrome and the nucleotide excision repair syndromes xeroderma pigmentosum, Cockayne syndrome and trichothiodystrophy (Levran et al, 1997). Each of these genes is responsible in a unique way for the integrity of the genome, and when mutated causes predisposition to cancer. FA patients are known to be at high risk for malignancy (Alter, 1996), while the cancer risk in FA carriers has not been well studied as yet. Cultured FA cells show baseline chromosomal instability as well as increased levels of chromosomal aberrations induced by DNA cross-linking agents. Since tumour cells are characterized by chromosomal instability, *FAA* was postulated to be a candidate for the gene targeted by LOH of this region. In addition, the breast tumour suppressor genes *BRCA*1 and *BRCA*2 were shown to play a role in DNA repair (Scully et al, 1997; Patel et al, 1998).

This report shows that, although a specific set of 19 breast tumours with isolated LOH of 16q24.3 was screened for mutations in the *FAA* gene using three different approaches, SSCP on cDNA, SSCP on genomic DNA and Southern analysis, no tumour-specific mutations were found. The SSCP analysis on genomic DNA was identical to that used in a study on FA patients (Wijker et al, 1998). This study shows that the method is able to identify mutations and polymorphisms. Moreover, our analysis is corroborated by the identification of naturally occurring polymorphisms in the *FAA* coding sequencing. The high number of polymorphisms found is in concordance with the recently reported variability of the *FAA* gene (Levran et al, 1997).

The *FAA* gene is rich in *Alu* sequences (Ianzano et al, 1997) suggesting *Alu*-mediated recombination resulting in large genomic deletions might be an important mechanism for the generation of *FAA* mutations. Several such genomic deletions, which would be missed by SSCP analysis, have been observed in FA patients (Fanconi Anemia/Breast Cancer Consortium, 1996; Centra et al, submitted; Levran et al, submitted). Therefore, a subset of seven tumours and six breast tumour cell lines was tested by Southern analysis to identify large genomic rearrangements, but this did not result in the identification of such mutations in breast tumours.

In conclusion, the *FAA* gene has been excluded as the breast tumour suppressor gene on 16q24.3 targeted by LOH. Therefore, another gene in this region remains to be identified.

### ACKNOWLEDGEMENTS

AM-CJ and EWM are supported by the Dutch Cancer Society grant RUL95-1040. ADA is supported by National Institutes of Health grant HL32987.

*© Cancer Research Campaign 1999* 

### REFERENCES

Alter BP (1996) Fanconi's anemia and malignancies. *Am J Hematol* **53**: 99–110

Berx G, Cleton-Jansen AM, Nollet F, De Leeuw WJF, Van de Vijver MJ, Cornelisse C and Van Roy F (1995) E-cadherin is a tumour invasion suppressor gene mutated in human lobular breast cancers. *EMBO J* **14**: 6107–6115

Berx G, Cleton-Jansen AM, Strumane K, De Leeuw WJF, Nollet F, Van Roy F and Cornelisse C (1996) E-cadherin is inactivated in a majority of invasive human lobular breast cancers by truncation mutations throughout its extracellular domain. *Oncogene* **13**: 1919–1925

Cher ML, Ito T, Weidner N, Carroll PR and Jensen RH (1995) Mapping of regions of physical deletion on chromosome 16q in prostate cancer cells by fluorescence in situ hybridization (FISH). *J Urol* **153**: 249–254

Cleton-Jansen AM, Moerland EW, Kuipers-Dijkshoorn NJ, Callen DF, Sutherland GR, Hansen B, Devilee P and Cornelisse CJ (1994) At least two different regions are involved in allelic imbalance on chromosome arm 16q in breast cancer. *Genes Chrom Cancer* **9**: 101–107

Devilee P, Van Vliet M, van Sloun P, Kuipers Dijkshoorn N, Hermans J, Pearson PL and Cornelisse CJ (1991) Allelotype of human breast carcinoma: a second major site for loss of heterozygosity is on chromosome 6q. *Oncogene* **6**: 1705–1711

Dorion-Bonnet F, Mautalen S, Hostein I and Longy M (1995) Allelic imbalance stidu of 16q in human primary breast carcinomas using microsatellite markers. *Genes Chrom Cancer* **14**: 171–181

Fanconi Anemia/Breast Cancer Consortium (1996) Positional cloning of the Fanconi anaemia group A gene. *Nat Genet* **14**: 324–328

Gschwend M, Levran O, Kruglyak L, Ranade K, Verlander PC, Shen S, Faure S, Weissenbach J, Altay C, Lander ES, Auerbach AD and Botstein D (1996) A locus for Fanconi anemia on 16q determined by homozygosity mapping. *Am J Hum Genet* **59**: 377–384

Ianzano L, D'Apolito M, Centra M, Savino M, Levran O, Auerbach AD, Cleton-Jansen AM, Doggett NA, Pronk JC, Tipping AJ, Gibson RA, Mathew CG, Whitmore SA, Apostolou S, Callen DF, Zelante L and Savoia A (1997) The genomic organization of the Fanconi anemia group A (*FAA*) gene. *Genomics* **41**: 309–314

Iida A, Yoshimoto M, Kasumi F, Nakamura Y and Emi M (1997) Localization of a breast cancer tumour-suppressor gene to a 3-cM interval within chromosomal region 16q22.1. *Br J Cancer* **75**: 264–267

Latil A, Cussenot O, Fournier G, Driouch K and Lidereau R (1997) Loss of heterozygosity at chromosome 16q in prostate adenocarcinoma: identification of three independent regions. *Cancer Res* **57**: 1058–1062

Levran O, Erlich T, Magdalena N, Gregory JJ, Batish SD, Verlander PC and Auerbach AD (1997) Sequence variation in the Fanconi anemia gene *FAA*. *Proc Natl Acad Sci USA* **94**: 13051–13056

Lo Ten Foe JR, Rooimans MA, Bosnoyan Collins L, Alon N, Wijker M, Parker L, Lightfoot J, Carreau M, Callen DF, Savoia A, Cheng NC, van Berkel CG, Strunk MH, Gille JJ, Pals G, Kruyt FA, Pronk JC, Arwert F, Buchwald M and Joenje H (1996) Expression cloning of a cDNA for the major Fanconi anaemia gene, *FAA*. *Nat Genet* **14**: 320–323

Moerland EW, Breuning MH, Cornelisse CJ and Cleton-Jansen A-M (1997) Exclusion of *BBC*1 and *CMAR* as candidate breast tumour suppressor genes. *Br J Cancer* **76**: 1550–1553

Patel KJ, Yu VPCC, Lee H, Corcoran A, Thistlethwaite FC, Evans MJ, Colledge WH, Friedman LS, Ponder BAJ and Venkitaraman AR (1998) Involvement of Brca2 in DNA repair. *Mol Cell* **1**: 347–357

Pronk JC, Gibson RA, Savoia A, Wijker M, Morgan NV, Melchionda S, Ford D, Temtamy S, Ortega JJ, Jansen S, Havenga C, Cohn RJ, De Ravel TJ, Roberts I, Westerveld A, Easton DF, Joenje H, Mathew CG and Arwert F (1995) Localisation of the Fanconi anaemia complementation group A gene to chromosome 16q24.3. *Nat Genet* **11**: 338–340

Scully R, Chen J, Plug A, Xiao Y, Weaver D, Feunteun J, Ashley T, Livingston DM (1997) Association of BRCA1 with Rad51 in mitotic and meiotic cells. *Cell* **88**: 265–275

Skirnisdottir S, Eiriksdottir G, Baldursson T, Barkardottir RB, Egilsson V and Ingvarsson S (1995) High frequency of allelic imbalance at chromosome region 16q22–23 in human breast cancer: correlation with high PgR and low S phase. *Int J Cancer* **64**: 112–116

Suzuki H, Komiya A, Emi M, Kuramochi H, Shiraishi T, Yatani R and Shimazaki J (1996) Three distinct commonly deleted regions of chromosome arm 16q in human primary and metastatic prostate cancers. *Genes Chrom Canc* **17**: 225–233

Tsuda H, Callen DF, Fukutomi T, Nakamura Y and Hirohashi S (1994) Allele loss on chromosome 16q24-qter occurs frequently in breast cancers irrespective of differences in phenotype and extent of spread. *Cancer Res* **54**: 513–517

Wijker M, Morgan NV, Herterich S, van Berkel CGM, Tipping AJ, Gross HJ, Gille JJP, Pals G, Savino M, Altay C, Mohan S, Dokal I, Cavenagh J, Marsh J, van Weel M, Ortega JJ, Schuler D, Samochatova E, Karwachi M, Bekassy AN, Abecasis M, Ebell W, Kwee ML, de Ravel T, Gibson RA, Gluckman E, Arwert F, Joenje H, Savoia A, Hoehn H, Pronk JC and Mathew CG (1998) Heterogenous spectrum of mutations in the Fanconi anaemia group A gene. *Eur J Human Genet* (in press)

*© Cancer Research Campaign 1999*
